# What every intensivist should know about using high-flow nasal oxygen for critically ill patients

**DOI:** 10.5935/0103-507X.20170060

**Published:** 2017

**Authors:** Martin Dres, Alexandre Demoule

**Affiliations:** 1 Sorbonne Universités, UPMC Univ Paris 06, INSERM, UMRS1158 Neurophysiologie Respiratoire Expérimentale et Clinique, Paris, France.; 2 AP-HP, Service de Pneumologie et Réanimation Médicale, Département "R3S", Groupe Hospitalier Pitié-Salpêtrière Charles Foix, Paris, France.

## Introduction

The most conventional forms of oxygen delivery rely on facemasks, a nasal cannula or nasal prongs. However, the use of these methods is limited by certain drawbacks, including the need for a flow of oxygen higher than 15L/min in case of severe hypoxemia or the dilution of administered oxygen by entrained room air in cases of high inspiratory flow. An alternative to conventional oxygen therapy has received growing attention: heated, humidified, high flow nasal oxygen (HFNO), a technique that can deliver heated and humidified oxygen, with a controlled fraction of inspired oxygen (FiO_2_), at a maximum flow rate of 60L/min via a nasal cannula. For a decade, the use of HFNO has been considered for patients with hypoxemic *de novo* acute respiratory failure (ARF). Recent reports suggest that HFNO can also be used to secure intubation and to prevent post-extubation ARF. The purpose of the present review is to provide clinicians with the most recent information on HFNO and to discuss its benefits and risks in its most common indications.

## How does high flow nasal oxygen work?

In physiological terms, HFNO improves the fraction of inspired oxygen, washes and reduces dead space, generates positive end-expiratory pressure (PEEP) and provides more comfort than cold and dry oxygen.

Patients with ARF have high inspiratory flow rates ranging between 30 and 120L/min. This flow commonly exceeds the maximum 15L/min flow rate that usual devices deliver. Subsequently, entrained room air dilutes the oxygen, which in turn decreases the FiO_2_. By delivering up to 100% oxygen at a maximum flow rate of 60L/min, HFNO minimizes the entrainment of room air and subsequently increases the FiO_2_.

A high rate of airflow delivered directly to the naso-pharynx improves carbon dioxide clearance by flushing the expired carbon dioxide from the upper airway.^(1)^ Subsequently, the dead space attributable to the flush out volume is reduced, thereby improving alveolar ventilation.^(2)^ The reduction of dead space contributes to the observed decrease in both the respiratory rate and the work of breathing.^(3)^

High flow nasal oxygen generates a varying PEEP level.^(4-6)^ In healthy volunteers treated with HFNO with a closed mouth and a flow rate of 60L/min, the measured PEEP was as high as 7cmH_2_O.^(6)^ However, this level of PEEP can decrease easily, as soon as the mouth is opened. Each 10L/min increase in flow rate increases the mean airway pressure by 0.69cmH_2_O when subjects breathe with their mouths closed, and by 0.35cmH_2_O when they breathe with their mouths open.^(5)^

Furthermore, HFNO delivers a heated and humidified flow, providing more comfort than dry air.^(7)^ HFNO also increases the water content of mucus, which facilitates secretion removal and avoids desiccation and epithelial injury. Finally, compared to other devices such as non-invasive ventilation (NIV), the tolerance of HFNO may be higher due to its interface, as simple nasal prongs enable patients to speak, eat and drink.

## What benefits can we expect from high flow nasal oxygen?

The expected benefits of HFNO depend on its indication and the device to which HFNO is compared (Table 1). To date, the use of HFNO has been suggested in several indications, but few have been rigorously evaluated (Table 2). The two primary indications in terms of level of evidence are (1) to prevent intubation in patients with hypoxemic de novo ARF and (2) to prevent post extubation ARF and subsequent reintubation in the medical ICU or after surgery. Other important indications in which HFNO has been investigated but which lack clear evidence of clinical benefits are preoxygenation before the intubation of severely hypoxemic patients, de novo hypoxemic ARF in immunocompromised hosts and oxygenation to secure flexible bronchoscopy.

**Table 1:** When high flow nasal oxygen can and cannot be used

HFNO can be used with some benefits	Hypoxemic acute respiratory failure without extrapulmonary organ failure
	After extubation in low risk patients
	Patients with do-not-intubate orders
HFNO can be used without clear benefits	After extubation following cardiothoracic surgery
	With flexible bronchoscopy
HFNO cannot be used	Hypoxemic ARF with criteria for intubation
	Hypoxemic ARF with extrapulmonary organ failure
Settings where HFNO use requires further clarification	Acute exacerbation of COPD
	Immunocompromised patients with ARF
	Preoxygenation for the intubation of hypoxemic patients
	Post extubation in surgical patients

HFNO - high flow nasal oxygen; ARF - acute respiratory failure; COPD - chronic obstructive pulmonary disease.

**Table 2:** Studies investigating high flow nasal oxygen in various intensive care unit settings

Studies	Design	Patients	HFNO was compared to	Primary endpoint	Results
Hypoxemic ARF					
Frat et al.^(8)^	RCT	310 Medical ICU	NIV and standard oxygen	Intubation	Similar but lower rates in the subgroup of patients with a PaO_2_/FiO_2_ < 200mmHg.
Post-surgery					
Stéphan et al.^(16)^	RCT	830 cardiothoracic surgery	Post-extubation NIV	Treatment failure	Non-inferiority
Futier et al.^(17)^	RCT	220 major abdominal surgery	Standard oxygen	Hypoxemia	No difference
Pre-intubation					
Miguel-Montanes et al.^(20)^	Before-After	101 medical ICU	Bag reservoir facemask	Lowest SpO_2_ during intubation	High SpO_2_ with HFNO
Semler et al.^(23)^	Open label	150 medical ICU	Usual care	Lowest SpO_2_ during intubation	No difference
Vourc’h et al.^(21)^	RCT	124 medical ICU	Oxygen facial mask	Lowest SpO_2_ during intubation	No difference
Jaber et al.^(22)^	RCT	49 medical ICU	HFNO + NIV *versus* NIV alone	Lowest SpO_2_during intubation	Higher SpO_2_ with HFNO + NIV
Post-extubation					
Maggiore et al.^(10)^	RCT	105 medical ICU	Venturi mask	PaO_2_/FiO_2_ ratio	Higher with HFNO
Hernández et al.^(11)^	RCT	527 low risk of post-extubation ARF medical ICU	Venturi mask	Reintubation	Lower with HFNO
Hernández et al.^(12)^	RCT	604 medical ICU with low risk of post-extubation ARF	NIV	Reintubation	No difference
Immunocompromised					
Lemiale et al.^(18)^	RCT	100 medical ICU	Standard oxygen	Need for NIV and/or intubation	No difference
Frat et al.^(19)^	RCT	82 medical ICU	NIV and standard oxygen	Intubation rate	Lower with HFNO

HFNO - high flow nasal oxygen; ICU - intensive care unit; RCT - randomized controlled trial; NIV - noninvasive ventilation; ARF - acute respiratory failure; PaO_2_/FiO_2_ - ratio of the partial pressure of arterial oxygen to the fraction of inspired oxygen; SpO_2_ - oxygen saturation level.

## In hypoxemic acute respiratory failure, high flow nasal oxygen may produce a lower intubation rate than standard oxygen and non-invasive ventilation in the most severely hypoxemic patients

The largest known study on HFNO in patients with hypoxemic ARF was recently conducted by Frat et al.^(8)^ This randomized controlled trial included patients with hypoxemic ARF and a ratio of partial pressure of arterial oxygen to the fraction of inspired oxygen (PaO_2_/FiO_2_) of 300mmHg or less, mainly due to pneumonia. Patients were assigned to receive HFNO, standard oxygen therapy delivered through a facemask, or NIV. The primary outcome was the rate of endotracheal intubation, which was similar among the three groups (38% in HFNO *versus* 47% in conventional oxygen therapy and 50% in NIV). However, in a post hoc analysis restricted to patients with severe initial hypoxemia defined by a PaO_2_/FiO_2_ ≤ 200mmHg, the intubation rate was significantly lower in patients who received high flow oxygen than in the other two groups. Furthermore, mortality, which was a secondary outcome, was significantly lower in the HFNO group than in the other two groups. Although these results are promising, they need to be confirmed by further trials before the systematic use of HFNO in patients with ARF and severe hypoxemia can be recommended.

## High flow nasal oxygen is a promising approach to prevent reintubation, but it should be used carefully in high-risk patients

Post extubation is a hazardous period for ICU patients and ARF after planned extubation is associated with remarkably high mortality.^(9)^ In this setting, ARF is related to many factors such as excessive secretions, progressive exhaustion, respiratory muscle weakness, aspiration or fluid overload. A first open label trial comparing HFNO to the use of a Venturi mask for 48 hours postextubation in patients with hypoxemia, but not ARF, led to reports of greater comfort, fewer desaturations, better interface tolerance and a lower reintubation rate with HFNO.^(10)^ In low-risk patients, HFNO applied for 24 hours postextubation compared to a Venturi mask was associated with a significant reduction in the need for reintubation.^(11)^ In high-risk patients, HFNO compared to NIV was not inferior to NIV to prevent reintubation and post extubation ARF.^(12)^

## High flow nasal oxygen is a valuable alternative to non-invasive ventilation to prevent acute respiratory failure in the post-surgical setting

Hypoxemia frequently occurs soon after major surgery,^(13,14)^ and expiratory complications are the second most frequent complications after surgery.^(15)^ In patients undergoing cardiothoracic surgery, NIV has been shown to prevent postoperative ARF. A multicenter randomized controlled trial has compared HFNO to NIV to treat post-extubation ARF or to prevent the occurrence of ARF in patients deemed at-risk.^(16)^ High flow nasal oxygen was not inferior to NIV, with a similar rate of treatment success. ICU mortality was similar in the two groups. A second large randomized controlled trial compared HFNO to standard oxygen in patients undergoing major elective abdominal surgery and deemed at moderate to high risk of postoperative pulmonary complications.^(17)^ The occurrence of postoperative hypoxemia one hour after extubation, of pulmonary complications and the length of hospital stays were similar between the two groups.

The benefit of HFNO in the postoperative setting is not unequivocal. Future studies should aim to identify the subset of patients who are the best candidates to benefit from HFNO.

## Other settings in which high flow nasal oxygen could be beneficial but require further investigation

High flow nasal oxygen has been proposed as an alternative to NIV to prevent intubation in hypoxemic de novo ARF in immunocompromised patients. To date, two post hoc analyses of large multicenter randomized controlled trials have been released, with conflicting results.^(18,19)^

High flow nasal oxygen has also been proposed as an alternative to NIV to prevent significant desaturation during endotracheal intubation in patients with severe hypoxemic ARF. Due to population heterogeneity, the results of these studies are conflicting,^(20-23)^ and additional studies targeting more homogeneous populations are needed.

Finally, HFNO may be useful for providing adequate oxygenation and comfort to end-of-life patients with do-not-intubate orders,^(24)^ but further studies are needed.

## Which precautions should be taken when using high flow nasal oxygen?

Clinicians should be aware of the potential harmful effects of HFNO. First, HFNO can mask patient worsening and subsequently delay intubation, which may be harmful. As recently reported by an observational study in de novo ARF patients treated by HFNO, patients intubated after more than 48 hours of treatment had a higher mortality than those intubated within 48 hours.^(25)^ In *de novo* hypoxemic ARF, HFNO preserves spontaneous breathing, permitting highly negative intrathoracic pressure. Therefore, HFNO can theoretically contribute to lung injury in patients breathing with high drive and large tidal volume.^(26)^ As opposed to NIV, no monitoring of pressures or volume is available for patients breathing with HFNO.

Clinicians willing to use HFNO in ICU patients need to implement the technique cautiously, carefully, and progressively in their unit, as with any new therapy. Identifying the patients who are the most likely to benefit from HFNO is a challenge.

## Conclusion

Over the past several years, a growing number of studies have suggested the potential benefit of HFNO in preventing intubation or reintubation in ICU patients who are either admitted for de novo acute respiratory failure or mechanically ventilated for surgery. Although HFNO appears to be a promising therapy in the ICU, additional studies are needed to define more precisely the subgroups of patients who are most likely to benefit from HFNO. Clinicians willing to use HFNO should know that HFNO might have deleterious effects, especially if it is not used adequately. As with any novel therapy, clinicians should learn how to use and implement HFNO progressively and cautiously.
